# 18F-Fluorodeoxyglucose Positron Emission Tomography/Computed Tomography Imaging in a Patient with HIV (-) Kaposi Sarcoma

**DOI:** 10.4274/mirt.07078

**Published:** 2016-09-29

**Authors:** Arzu Cengiz, Ekin Şavk, Canten Tataroğlu, Yakup Yürekli

**Affiliations:** 1 Adnan Menderes University Faculty of Medicine, Department of Nuclear Medicine, Aydın, Turkey; 2 Adnan Menderes University Faculty of Medicine, Department of Dermatology, Aydın, Turkey; 3 Adnan Menderes University Faculty of Medicine, Department of Pathology, Aydın, Turkey

**Keywords:** positron emission tomography/computed tomography, kaposi sarcoma, HIV

## Abstract

Kaposi sarcoma (KS) is a vascular neoplasm that often manifests with multiple vascular nodules on the skin and other organs. Various imaging modalities can be used to display disease extent. Herein we present a 65-year-old female patient with human immunodeficiency virus negative KS along with her whole-body positron emission tomography/computed tomography imaging findings.

## Abstract (tr)

Kaposi sarkomu (KS), deri ve diğer organlarda yaygın vasküler nodüllerle ortaya çıkan vasküler bir tümördür. Hastalığın yaygınlığını göstermek için çeşitli görüntüleme modaliteleri kullanılabilmektedir. Bu çalışmada 65 yaşında insan bağışıklık yetmezlik virüsü negatif KS olan bir kadın hastayı ve bu hastanın tüm vücut pozitron emisyon tomografi/bilgisayarlı tomografi görüntüleme bulgularını sunduk.

## INTRODUCTION

Kaposi sarcoma (KS) is an immunodeficiency syndrome-related disease that has been reported to be strongly associated with human herpes virus-8 (1,2). The skin, mucosal surfaces and lung are the main sites of involvement. Visceral involvement predicts survival especially in patients with acquired immune deficiency syndrome (AIDS)-associated KS, thus accurate staging and identification of diseased sites with fluorodeoxyglucose (FDG) positron emission tomography/computed tomography (PET/CT) is useful in the management of these patients (3). Herein we present a patient with human immunodeficiency virus (HIV)-negative KS staged by 18F-FDG PET/CT imaging.

## CASE REPORT

**Figure 1:**
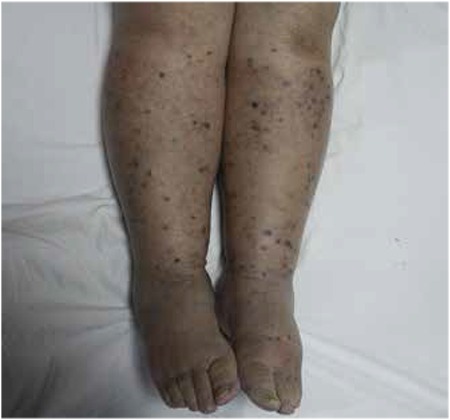
Dark blue-purplish macular and nodular skin lesions on the legs

**Figure 2:**
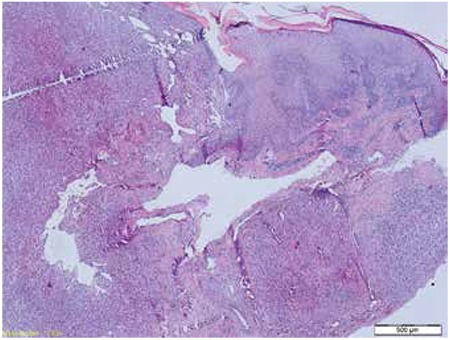
The underlying epidermis spindled cells showing lobular growth pattern (hematoxylin and eosin x40)

**Figure 3:**
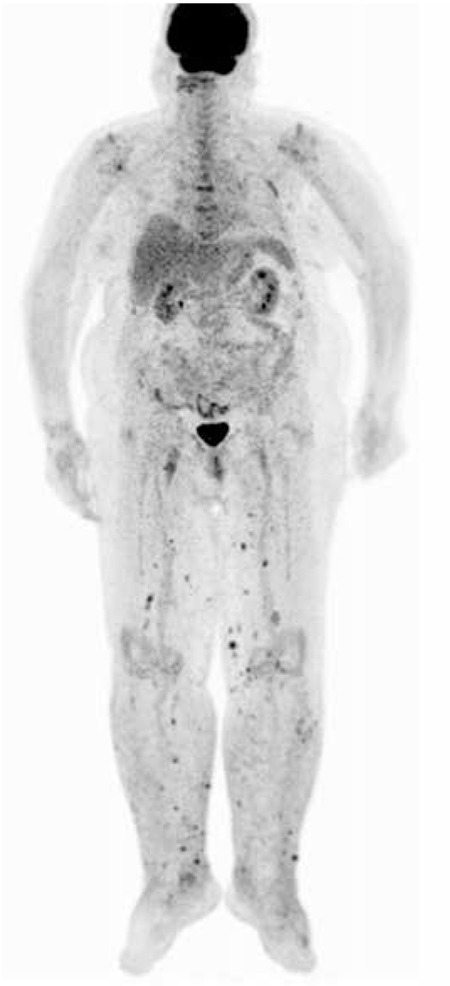
Maximum intensity projection images show multiple nodular lesions with increased fluorodeoxyglucose uptake on the skin and subcutaneous tissues of the legs (SUV_max_: 6.1), and hypermetabolic lymph nodes at bilateral inguinal and popliteal sites

A 65-year-old female patient was referred to our hospital with complaints of swelling and nodular skin lesions on both legs. She had a history of rheumatoid arthritis and treatment with corticosteroid medication for five years. On physical examination, dark blue-purplish macular and nodular skin lesions were observed on the legs along with pretibial edema (Figure 1). The lesions that had appeared within a few months were not painful. She was diagnosed with KS with biopsy of the skin lesions (Figure 2). Laboratory tests were within normal limits except an elevated erythrocyte sedimentation rate. Anti-HIV antibody was negative. She was referred to our department for initial staging with ^18^F-FDG PET/CT imaging. A whole body ^18^F-FDG PET/CT imaging was performed 60 minutes after 370 megabequerel ^18^F-FDG injection using an integrated PET/CT scanner (Siemens, Biograph mCT, Germany). ^18^F-FDG PET/CT imaging showed multiple nodular skin lesions with increased FDG uptake on both legs (SUV_max_: 6.1). In addition, there were hypermetabolic bilateral inguinal and popliteal lymph nodes (SUV_max_: 3.6-5.6) (Figure 3).

## LITERATURE REVIEW AND DISCUSSION

KS is a common tumor in AIDS patients. Most patients present with a single or few lesions, however multiple lesions have also been reported (4). In most cases, the lesions are asymptomatic. Four variants of KS have been recognized clinically: classical KS, endemic (African) KS, iatrogenic (organ transplant-related) KS, and AIDS-related (epidemic) KS (5). Disease stage, clinical type and immune status are important in determining treatment options including surgery, radiotherapy, chemotherapy and immunotherapy. Various imaging modalities including gastrointestinal endoscopy, conventional radiography, CT, magnetic resonance imaging (MRI), and radionuclide imaging are used for staging. Imaging findings depend on the organ systems that are affected. CT and MRI are generally more useful in the assessment of visceral and lymphatic KS. Thoracic disease, which is a common visceral involvement, bilateral hilar lymphadenopathy and bilateral involvement in the mid and lower lung zones with peribronchial and perivascular opacities is characteristic on high resolution CT. MRI has higher sensitivity in detecting cardiac lesions and bone involvement (6,7).

Thallium-201 (^201^Tl) and gallium-67 (^67^Ga) scintigraphy had been previously used for differential diagnosis. ^67^Ga negative and ^201^Tl positive lesions were most likely accepted as KS, whereas both ^67^Ga and ^201^Tl positive lesions were considered as lymphoma (8). ^99m^Tc tetrofosmin had also been once used in patients with KS as a tumor screening agent (9).

Recently, ^18^F-FDG PET/CT imaging is being used for the evaluation of visceral and lymphatic involvement, and staging of KS. It has a role in both staging and the evaluation of response to therapy (10,11). In addition, PET/CT is an effective method in detecting clinically occult KS lesions that were not detected with other imaging methods (12). KS may demonstrate heterogeneous FDG avidity. In some previous studies, lymph node and visceral involvement such as the bone and lungs were detected by ^18^F-FDG PET/CT imaging (10,11,13,14). Diffuse and focal FDG uptake in the skin have also been reported (14,15). In our patient, an increased FDG uptake was detected in nodular skin lesions on the lower extremities, and the highest SUV_max_ value of these lesions was 6.1. ^18^F-FDG PET/CT imaging detected lymph node involvement in addition to widespread cutaneous involvement in our patient.

In conclusion, whole body ^18^F-FDG PET/CT imaging can detect the extent of visceral and lymphatic involvement, and makes a significant contribution in both staging and clinical management of KS.

## Ethics

Informed Consent: Consent form was obtained from all participants.

Peer-review: Externally peer-reviewed.

Financial Disclosure: The authors declared that this study has received no financial support.
